# Renal replacement therapy for critically ill patients: an intermittent continuity

**DOI:** 10.1186/cc13756

**Published:** 2014-03-05

**Authors:** Zaccaria Ricci, Stefano Romagnoli

**Affiliations:** 1 Department of Cardiology and Cardiac Surgery, Pediatric Cardiac Intensive Care Unit, Bambino Gesù Children’s Hospital, IRCCS, Piazza S. Onofrio 4, 00165 Rome, Italy; 2 Department of Health Sciences – Section of Anesthesiology and Intensive Care, University of Florence, Azienda Ospedaliero – Universitaria Careggi, Largo Giovanni Alessandro Brambilla 3, 50139 Florence, Italy

## Abstract

Choice of the right renal replacement therapy for severe acute kidney injury in critically ill patients has been investigated many times in the last two decades. Although some questions have been answered, in current practice many different approaches are still used in the ICU. One basic and important issue is the frequency of renal replacement delivery: apart from pathophysiological speculations, in terms of hard outcomes (namely mortality and length of hospital stay) should dialysis be delivered continuously or intermittently? The authors of the CONVINT study provided a (last) response to this debate: in expert hands, the two treatments provide similar outcomes. This study confirms previous studies and is also important for other aspects, such as the possibility that the two modalities are complementary and may be indicated for different purposes.

## 

The never-ending comparison between outcomes of intermittent and continuous renal replacement therapy (RRT) for renal support in the ICU has come to such a level of unprecedented and unequivocal evidence [1-4] that, currently, meeting organizers might stop including this topic in their programs. Nevertheless, many clinicians still have concerns regarding which technique to routinely apply to their patients. A recent trial by a multidisciplinary group of German physicians with specific expertise in critical care nephrology provided new data on this important field [1]. Indeed, Schefold and colleagues conducted the randomized controlled trial Effect of Continuous Versus Intermittent Renal Replacement Therapy on the Mortality and Outcome of Acute Renal Failure in ICU Patients (CONVINT) comparing daily hemodialysis and postdilution continuous hemofiltration, and confirmed that hard outcomes such as mortality, dialysis duration or length of hospital stay are not significantly affected by the choice of RRT modality (continuous or intermittent). They randomized very well-matched populations and delivered adequate and intense treatments in terms of dose prescription. Interestingly, the authors also detailed several important secondary outcomes, such as days on RRT, dialysis-free days, vasopressor days, days on ventilator as well as course of urinary output, serum creatinine and serum urea concentrations that were all similar in the two groups.

This trial confirmed another three important aspects. First, designing and conducting such a study is extremely difficult. As a matter of fact, the authors were compelled to stop enrolling patients before the estimated sample size was reached because technology and material for delivering continuous therapies was no longer available in their center. In contrast to a pharmacological intervention study, where the amount of study drugs may be stored before the trial is started, when a RRT device (or procedure) evaluation is planned the study may be concluded prematurely if the hospital stops supply of the device.

Second, the possibility of switching one randomized treatment to another cannot be excluded. The authors showed that switching of modality occurred in about 20% of intermittent hemodialysis (IHD) patients due to hemodynamic instability and/or significant fluid overload. In the continuous RRT group, switching of modality was indicated in 46% of cases because of repeated filter clotting, metabolic reasons, bleeding or issues with anticoagulation, thrombocytopenia or clinical improvement. The high rate of treatment switch was a key issue of Schefold and colleagues’ study. The possibility to switch from intermittent to continuous treatment and *vice versa* suggests that the two techniques may be seen as complementary rather than alternative. In other words, expert clinicians in routine clinical practice may conceive to combine the benefits of both modalities, tailoring RRT from patient to patient and from session to session.

Finally, the time has come to conduct an equivalence trial since no randomized controlled trial was able, so far, to convincingly show the superiority of one modality over the other: an important issue of equivalence trials, however, is the generally required large sample sizes [5]. The study by Schefold and coworkers was probably significantly underpowered to demonstrate such equivalence.

One can now conclude that no specific recommendation should be given about any superiority between IHD and continuous RRT. Ideally, both should be available in the ICU. On the other hand, it is our personal opinion that if a center/institution/department has developed a particular expertise with one modality, then an abrupt change to another may be detrimental and is not recommended: the best treatment may be the one that you know better. One last aspect has not to be forgotten, however: if short-term hard outcomes are not impacted by RRT modality, the situation may be different for long-term outcomes. It is noteworthy that IHD has been suspected to cause long-term chronic kidney disease in previously critically ill acute kidney injury patients. At least two recent persuasive studies launched an important alarm signal [6,7]: compared with continuous RRT, initiation of IHD in critically ill adults with acute kidney injury is associated with a higher likelihood of chronic dialysis. If this was confirmed, IHD as a first-line treatment in the ICU should be reserved for acute or chronic patients or for those who are less likely to be weaned from RRT.

In conclusion, during continuous RRT and in the absence of citrate anticoagulation [8] the circuit lifespan averages 20 hours, with downtime further shortening the daily treatment time [9]. On the other hand, one may speculate that softer and more prolonged intermittent dialytic sessions may reduce IHD side effects (especially hemodynamic instability) and improve long-term outcomes of acute kidney injury patients [10]. In the future, to achieve the best short-term to long-term outcomes, the two modalities may possibly meet in the middle field of intermittent continuity.

### Abbreviations

CONVINT: Effect of Continuous versus Intermittent Renal Replacement Therapy on the Mortality and Outcome of Acute Renal Failure in ICU Patients; IHD: Intermittent hemodialysis; RRT: Renal replacement therapy.

### Competing interests

The authors declare that they have no competing interests.
